# The enzyme activity of sortase A is regulated by phosphorylation in *Staphylococcus aureus*

**DOI:** 10.1080/21505594.2023.2171641

**Published:** 2023-02-10

**Authors:** Feifei Chen, Hongxia Di, Yanhui Wang, Chao Peng, Rongrong Chen, Huiwen Pan, Cai-Guang Yang, Haihua Liang, Lefu Lan

**Affiliations:** a College of Life Science, Northwest University, Xi’an, China; b State Key Laboratory of Drug Research, Shanghai Institute of Materia Medica, Chinese Academy of Sciences, Shanghai, China; c Hangzhou Institute for Advanced Study, University of Chinese Academy of Sciences, Hangzhou, China; d University of Chinese Academy of Sciences, Beijing, China; e National Facility for Protein Science in Shanghai, Zhangjiang Lab, Shanghai Advanced Research Institute, Chinese Academy of Science, Shanghai, China; f School of Medicine, Southern University of Science and Technology, Shenzhen, China

**Keywords:** *Staphylococcus aureus*, Sortase A, protein phosphorylation, virulence

## Abstract

In many Gram-positive bacteria, the transpeptidase enzyme sortase A (SrtA) anchors surface proteins to cell wall and plays a critical role in the bacterial pathogenesis. Here, we show that in *Staphylococcus aureus*, an important human pathogen, the SrtA is phosphorylated by serine/threonine protein kinase Stk1. *S. aureus* SrtA can also be phosphorylated by small-molecule phosphodonor acetyl phosphate (AcP) *in vitro*. We determined that various amino acid residues of *S. aureus* SrtA are subject to phosphorylation, primarily on its catalytic site residue cysteine-184 in the context of a bacterial cell lysate. Both Stk1 and AcP-mediated phosphorylation inhibited the enzyme activity of SrtA *in vitro*. Consequently, deletion of gene (i.e. *stp1*) encoding serine/threonine phosphatase Stp1, the corresponding phosphatase of Stk1, caused an increase in the phosphorylation level of SrtA. The *stp1* deletion mutant mimicked the phenotypic traits of *srtA* deletion mutant (i.e. attenuated growth where either haemoglobin or haem as a sole iron source and reduced liver infections in a mouse model of systemic infection). Importantly, the phenotypic defects of the *stp1* deletion mutant can be alleviated by overexpressing *srtA*. Taken together, our finding suggests that phosphorylation plays an important role in modulating the activity of SrtA in *S. aureus.*

## Introduction

*Staphylococcus aureus* is an important pathogen that causes life-threatening infections and poses a major public health threat due to the emergence of multidrug resistance [1]. Potential alternative approaches to the treatment of *S. aureus* infection, particularly those caused by Methicillin-resistant *S. aureus* (MRSA), are agents that target *S. aureus* virulence [2–5] and may include the inhibition of pathways such carotenoid biosynthetic pathway [6,7], accessory gene regulator (*agr*) [8–10], and sortase enzymes that anchor surface proteins to the cell wall [11–18].

Sortases are transpeptidases found in many Gram-positive bacteria [14,19,20]. Among them, *S. aureus* sortase A (SrtA) is the archetypal sortase that cleaves secreted protein substrates between the threonine and glycine residues of the conserved C-terminal LPXTG sorting motif and links the newly released threonine residue to the pentaglycine cross-bridge of *S. aureus* peptidoglycan, thereby effectively immobilizing substrates into the cell wall [14]. In *S. aureus*, 20 proteins with LPXTG sorting signals fulfill diverse functions such as iron acquisition from the host, bacterial adhesion, and immune evasion during the infectious process [14]. SrtA plays a critical role in the pathogenesis of Gram-positive bacterial infections and a *S. aureus srtA* knockout mutant is unable to cause bacteraemia or sepsis in mice [11,14,21].

SrtA is not required for bacterial viability *in vitro* and belongs to an exclusively bacterial family of enzymes, suggesting that specific SrtA inhibitors may have a limited selective pressure to promote the rise of resistance traits and are unlikely to exhibit particularly deleterious side effects [16]. SrtA is thus considered an ideal target for anti-virulence drug development [14–24]. The transpeptidase activity of SrtA has also been exploited in a range of industrial applications, including protein conjugation and targeted labelling of cell surface macromolecules [25–32]. To date, *S. aureus* SrtA is the most understood of all the sortases and its molecular features (*i.e*. structures, functions, and mechanisms) have been extensively studied [14,20] subsequent to its discovery in 1999 [11].

SrtA contain three conserved residues within its active site: His120, Cys184, and Arg197 [33]. It is clear that Cys184 forms a covalent linkage to the sorting signal while the functions of His120 and Arg197 are controversial [34]. Currently, the specificity and kinetic/catalytic mechanism of sortases remains an active area of research and a reverse protonation chemical mechanism of *S. aureus* SrtA has been proposed recently [34]. In this mechanism, C184 thiolate is the active site nucleophile, H120 imidazolium is a general acid, and R197 is a transition state stabilizer [34].

Although insight into the biological function, structure, specificity and mechanism of *S. aureus* SrtA is significant at present [14,20,34], the mechanisms that may enable *S. aureus* to regulate the activity of SrtA have not been described. Here, we show that *S. aureus* SrtA is subject to post-translational regulation. We found that phosphorylation markedly decreased the enzyme activity of *S. aureus* SrtA. We also showed that SrtA is a key factor for the Stk1/Stp1 signalling pathway in *S. aureus*.

## Results

### SrtA is a substrate for Stk1 *in* *vitro*

**Figure 1.**
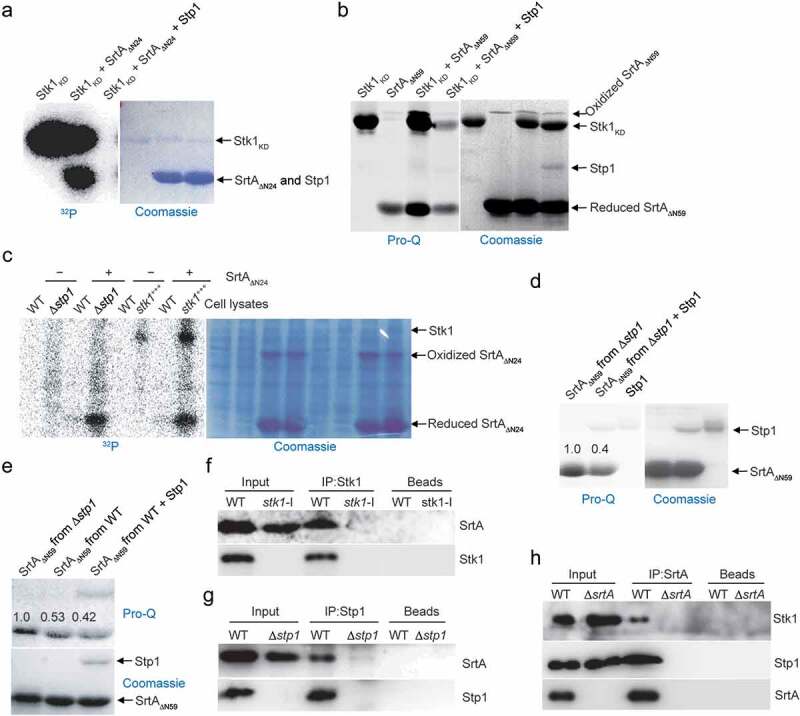
Reversible phosphorylation of SrtA and Co-IP experiments. (**A**) Recombinant SrtA_ΔN24_ was incubated with the recombinant Stk1_KD_ in the presence of [γ-^32^P]ATP. Stp1 proteins were added subsequent to the *in vitro* phosphorylation reaction in order to dephosphorylate SrtA_ΔN24_ as indicated. Recombinant SrtA_ΔN24_ and Stp1 have similar molecular weights (~27 KD) and migrate similarly in SDS-PAGE. (**B**) Reversible phosphorylation of SrtA by Stk1 and Stp1. (**C**) Phosphorylation of SrtA_ΔN24_ in the context of a bacterial lysate. WT, cell lysates from wild-type Newman strain (harbouring pYJ335); Δ*stp1*, cell lysates from a Δ*stp1* strain (harbouring pYJ335); *stk1*^+++^, cell lysates from *S. auerus* strain overexpressing the *stk1* (Newman/pYJ335:*stk1*). (**D** and **E**) Dephosphorylation of recombinant SrtA_ΔN59_ expressed and purified from either Δ*stp1* or WT Newman strain, band intensities from Pro-Q Diamond chemiluminescence were normalized against the corresponding coomassie brilliant blue stained band, which were set to 1.0 for purified recombinant SrtA_ΔN59_ from the Δ*stp1* strain. In **A-E**, Reactions were stopped by addition of SDS – PAGE loading buffer (without DTT), samples were separated by SDS-PAGE, visualized by either autoradiography (^32^P) or pro-Q diamond stained gel (Pro-Q), and stained with coomassie blue. (**F**) Co-IP between Stk1 and SrtA. IP was performed with anti-Stk1 antibody followed by immunoblotting with anti-SrtA antibody (SrtA) and anti-Stk1 antibody (Stk1). The beads control was obtained in the absence of Stk1 antibody. (**G**) Co-IP between Stp1 and SrtA. IP was performed with anti-Stp1 antibody followed by immunoblotting with anti-SrtA antibody (SrtA) and anti-Stp1 antibody (Stp1). The beads control was obtained in the absence of Stp1 antibody. (**H**) Co-IP between SrtA and Stk1 or Stp1. IP was performed with anti-SrtA antibody followed by immunoblotting with anti-Stk1 antibody (Stk1), anti-Stp1 antibody (Stp1), and anti-SrtA antibody (SrtA). The beads control was obtained in the absence of SrtA antibody. In **F-H**, WT denotes WT *S. aureus* Newman strain, Δ*stp1* denotes *stp1* deletion mutant, *stk1*-I denotes *S. aureus* Newman mutant with transposon insertion in *stk1* gene, Δ*srtA* denotes *srtA* deletion mutant.

Eukaryote-type Ser/Thr protein kinases (STKs) and phosphatases (STPs) have emerged as critical signalling molecules in prokaryotes [35,36]. In *S. aureus*, Stk1 and Stp1 provide an additional level of regulation for a variety of biological functions, including cell wall biosynthesis, drug resistance and virulence regulation [36–39]. To examine whether SrtA is a substrate for Stk1, the catalytic domain of *S. aureus* Stk1, termed Stk1_KD_, was cloned, expressed, and purified from *E. coli*. Using SDS-PAGE and autoradiography, we found that SrtA_ΔN24_, a truncated tagged form of *S. aureus* SrtA [40], was phosphorylated by Stk1_KD_ *in vitro* (Figure 1A). Similar results were obtained using the Pro-Q Diamond and Coomassie blue stains with purified SrtA_ΔN59_ (Figure 1B), another truncated *S. aureus* SrtA lacking the N-terminal 59 residues [40]. In the presence of Stk1_KD_, the SrtA_ΔN59_ band stained intensely with Pro-Q Diamond but not with Coomassie blue (Figure 1B). These results clearly suggest that SrtA is a substrate of Stk1 *in vitro*. Either phosphorylated SrtA_ΔN24_ (Figure 1A) or phosphorylated SrtA_ΔN59_ (Figure 1B) was dephosphorylated by Stp1, indicating that phosphorylated SrtA is a substrate of Stp1.

To further examine the roles of Stk1 and Stp1 in the phosphorylation of SrtA, we performed phosphorylation assays by incubating SrtA_ΔN24_ proteins respectively with cell lysates of a wild-type (WT) Newman strain, a Δ*stp1* stain, and a Stk1-overproducing strain (*stk1*^+++^, the WT Newman strain carrying pYJ335:*stk1*). When cell lysates of either Δ*stp1* or *stk1*^+++^ strain was used, a higher phosphorylation level of SrtA_ΔN24_ was observed compared to that obtained using the cell lysates of WT Newman strain (Figure 1C). Thus, both *stp1* deletion and over-expression of *stk1* facilitate the phosphorylation of SrtA in the context of a cell lysate. These results are also consistent with *in vitro* reversible phosphorylation of SrtA mediated by purified recombinant Stk1 and Stp1 (Figure 1A and 1B).

## Phosphorylation of recombinant SrtA expressed in *S. aureus*

To probe the existence of phosphorylation of SrtA *in vivo*, we purified SrtA_ΔN59_ from Δ*stp1* and WT *S. aureus* Newman strain respectively, and dephosphorylation assay was performed by incubating the SrtA_ΔN59_ protein with Stp1. As shown in (Figure 1D), Stp1-treatment decreased the intensity of SrtA_ΔN59_ band stained with Pro-Q Diamond but not with Coomassie blue, indicating that SrtA is phosphorylated *in vivo*. SrtA_ΔN59_ proteins purified from the Δ*stp1* strain displayed higher intensity of Pro-Q Diamond stained band than those purified from the WT Newman strain (Coomassie staining as loading control) (Figure 1E). We also noted that Stp1-treatment decreases the phosphorylation level of SrtA_ΔN59_ purified from the WT Newman strain (Figure 1E), but to a much lesser extent than it did for the SrtA_ΔN59_ that had been purified from the Δ*stp1* strain (Figure 1D). Based on these data, we conclude that SrtA is very likely to be subject to reversible phosphorylation *in vivo*, and this effect appears to be mediated by at least Stk1 and Stp1. Supporting this view, Co-Immunoprecipitation (Co-IP) assays demonstrated the interactions between SrtA and Stk1, and between SrtA and Stp1 (Figure 1F toH).

## SrtA contains multiple phosphorylation sites

**Figure 2.**
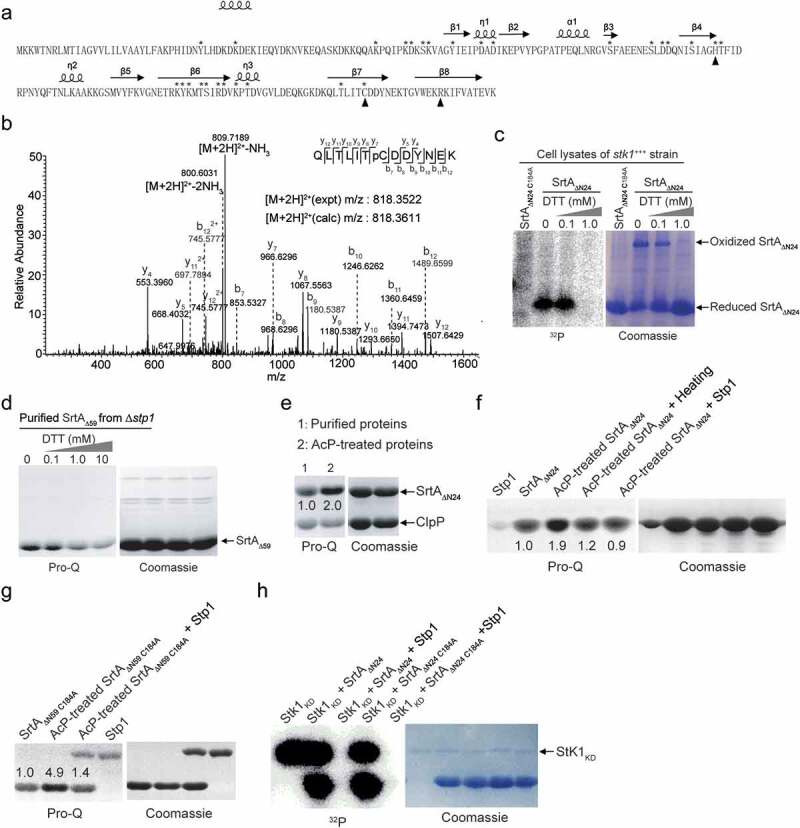
Different phosphorylation events on SrtA. (**A**) Phosphorylation sites of SrtA revealed by mass spectrometry (MS/MS). Phosphorylation sites are highlighted with asterisk, the catalytic residues of SrtA (Cys184, His120, and Arg197) are highlighted with triangle, and some secondary structural elements are indicated according to the structure of *S. aureus* SrtA (PDB ID 1T2P). (**B**) A representative MS/MS spectrum of phosphorylation on the Cys184 of SrtA. The phosphor-peptide QLTLITpCDDYNEK was observed at m/z 818.3611 Da obtained after trypsin digestion of SrtA that has been phosphorylated by the bacterial cell lysates of *stk1*^+++^ strain. Individual fragments are labelled based on the b- or y-ion nomenclature. (**C**) *In vitro* phosphorylation of SrtA_ΔN24_ and its variant by bacterial cell lysates of *stk1*^+++^ strain in the presence of [γ-^32^P]ATP. *stk1*^+++^, *stk1*-overexpressing strain (Newman/pYJ335:*stk1*). SrtA_ΔN24 C184A_, variant of SrtA_ΔN24_ harbouring a Cys to Ala substitution at C184. (**D**) Effect of DTT on the phosphorylation of SrtA that has been purified from Δ*stp1* strain. (**E**) Effect of AcP (25 mM) on the phosphorylation of SrtA_ΔN24_ and ClpP *in vitro*. **(F)** Effect of sample heating or Stp1-treatment on the AcP-mediated phosphorylation of SrtA_ΔN24_. **(G)** SrtA_ΔN59 C184A_ could be phosphorylated by acetyl phosphate (25 mM). (**H**) Phosphorylation of SrtA_ΔN24 C184A_ by Stk1. SrtA_ΔN24_ and SrtA_ΔN24 C184A_ were respectively incubated with recombinant Stk1_KD_ in the presence of [γ-^32^P]ATP; Stp1 was added subsequent to *in vitro* phosphorylation reaction in order to dephosphorylate SrtA_ΔN24_ and SrtA_ΔN24 C184A_. The reactions were stopped by addition of SDS – PAGE loading buffer (without DTT). Samples were separated by SDS-PAGE, visualized by autoradiography (^32^P) or Pro-Q diamond stained gel (Pro-Q), and stained with coomassie blue (Coomassie). recombinant SrtA_ΔN24_ and Stp1 have similar molecular weights (~27 KD) and migrate similarly. SrtA_ΔN24 C184A_, SrtA_ΔN24_ variant harbouring a Cys to Ala substitution at C184.

To further verify the phosphorylation of SrtA and to identify the phosphorylation sites, we performed mass spectrometric characterization on the SrtA protein phosphorylated by the cell lysates of *stk1*^+++^ strain. Searching tandem mass spectrometry (MS/MS) spectra for neutral loss peaks of 80 daltons revealed the presence of at least 28 phosphorylation sites and that the SrtA protein can be phosphorylated simultaneously at multiple residues (Figure 2A, Fig. S1 A to E, and Table S3). These phosphorylation sites are mainly located in and around strands ß1, ß3 and ß4, strand ß6 and several amino acid residues close to the end of it, and strand ß7 of the crystal structure of SrtA (Figure 2A) [41]. The catalytic residues of *S. aureus* SrtA, Cys184, and His120 [14], were subject to phosphorylation (Figure 2B, Fig. S1). Asp112, which is important for calcium binding [42] and SrtA activity [43], was also a potential phosphorylation site (Figure 2A, Table S3). These observations indicate that SrtA contains multiple phosphorylation sites.

## Cysteine phosphorylation of SrtA

As aforementioned, Cys184 of *S. aureus* SrtA was subject to phosphorylation (Figure 2B). Because Cys184 is essential for the catalytic activity of SrtA and this residue is absolutely conserved and essential for sortase activity from Gram-positive bacteria [14], we next sought to determine the role of Cys184 in the phosphorylation of SrtA. To this end, we analysed the phosphorylation levels of SrtA_ΔN24_ and its mutant protein SrtA_ΔN24 C184A_ (in which Cys184 was replaced by an alanine residue) in the cell lysates of *stk1*^+++^ strain in the presence of [^32^P] ATP using autoradiography. Our results showed that phosphorylation occurred exclusively to the monomeric reduced forms of SrtA_ΔN24_, but not to its dimeric oxidized forms and its mutant forms (SrtA_ΔN24 C184A_) (Figure 2C). These observations suggest that Cys184 might be a key site responsible for the phosphorylation of SrtA in the context of a bacterial cell lysate.

Additionally, we observed that, in the presence of 1 mM dithiothreitol (DTT), phosphorylation of SrtA_ΔN24_ by the cell lysates was almost abolished (Figure 2C). This result support the notion that Cys184 was very likely the major site of the observed phosphorylation (Figure 2C, lane 2) since cysteine phosphorylation is DTT-reversible [36]. Using Pro-Q Diamond and Coomassie blue stains, we found that the phosphorylation of SrtA_ΔN59_ that had been purified from Δ*stp1* stain displayed liability towards DTT (Figure 2D). Taken together, these biochemical data, along with the LC-MS/MS data (Figure 2B), suggest that Cys184 is a primary site for the phosphorylation of SrtA in the context of a bacterial cell lysate and cellular.

## Different phosphorylation events on SrtA

Since several aspartic acid residues of *S. aureus* SrtA were subject to phosphorylation (Figure 2A, Fig. S1, and Table S3), we next sought to analyse the effect of acetyl phosphate (AcP) on the phosphorylation of SrtA, given that AcP can be used as a phosphodonor for the acceptor aspartic acid residue of some bacterial proteins such as response regulators [44–49]. Using Pro-Q Diamond and Coomassie blue stains, we found that SrtA_ΔN24_, but not Caseinolytic protease proteolytic subunit (ClpP) that serves as a control, can be efficiently phosphorylated by AcP (Figure 2E). We also observed that sample heating, which promotes extensive hydrolysis of phospho-Asp residues [50], caused essentially loss of phosphorylation of SrtA_ΔN24_ that has been treated by AcP (Figure 2F). These results indicate that certain aspartic acid residue of *S. aureus* SrtA may serve as acceptor for the phosphodonor AcP. Additionally, Stp1-treatment also decreased the phosphorylation level of SrtA_ΔN24_ mediated by AcP (Figure 2F), indicating that Stp1 may possess aspartic acid dephosphorylation activity.

**Figure 3.**
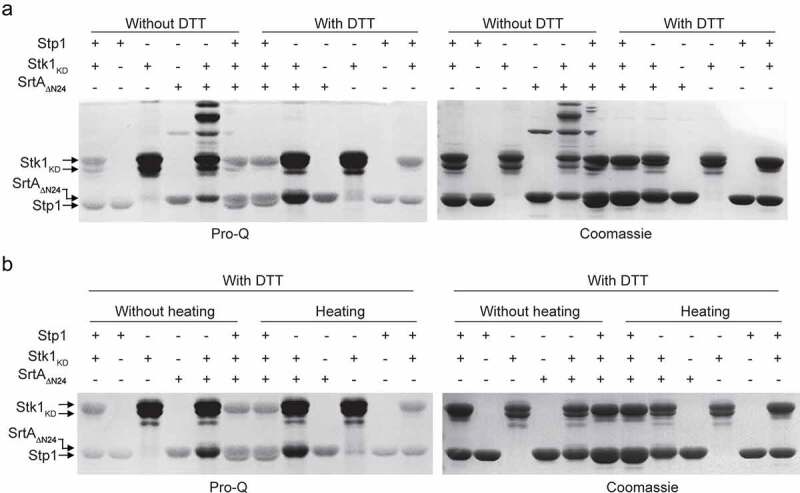
*In vitro* phosphorylation of SrtA_ΔN24_. (**A**) Effect of DTT on Stk1-mediated phosphorylation of SrtA_ΔN24_. The reaction was stopped by addition of SDS-PAGE loading buffer containing without or with DTT at a final concentration of 50 mM. samples were separated by SDS – PAGE, visualized by pro-Q diamond stained gel (left panel), and stained with Coomassie Blue (right panel). (B) Effect of sample heating on Stk1-mediated phosphorylation of SrtA_ΔN24_. The reaction was stopped by addition of SDS – PAGE loading buffer (with DTT at a final concentration of 50 mM) without or with sample heating (95 °C, 10 min). samples were separated by SDS – PAGE, visualized by pro-Q diamond stained gel (left panel), and stained with coomassie blue (right panel).

AcP was able to efficiently phosphorylate not only the SrtA_ΔN24_ (Figure 2E andF) but also the SrtA_ΔN59 C184A_ (Figure 2G). Additionally, the cysteine residue does not play an important role in the *in vitro* phosphorylation of SrtA by purified Stk1_KD_ (Figure 2H). Thus, the Cys184 is unlikely a primary phosphorylation site of SrtA under these experimental conditions, although it did in the context of a bacterial cell lysate (Figure 2C) and cellular (Figure 2D). Moreover, we observed that phosphorylated SrtA_ΔN24_ that had been phosphorylated by purified Stk1_KD_ was not susceptible to either DTT treatment (Figure 3A) or sample heating (Figure 3B). In all, these data suggest that SrtA can contain different types of phosphorylation with distinct chemical properties including DTT-labile (Figure 2C and 2D), heat-labile (Figure 2F), DTT-stable (Figure 3A), and heat-stable (Figure 3B). These distinct chemical properties are probably due to phosphorylation occurring on different forms of amino acid residues, which is in line with the observation that various forms of amino acid residues of SrtA were phosphorylatable (Figure 2A, Fig. S1, and Table S3).

## Phosphorylation inhibits the enzymatic activity of SrtA

**Figure 4.**
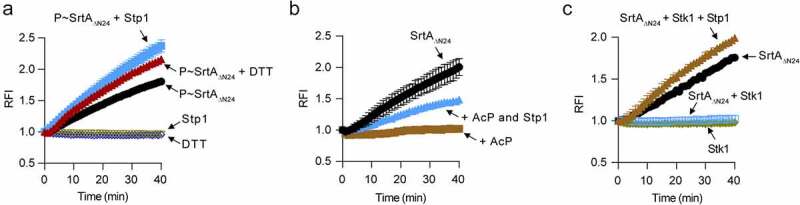
Phosphorylation inhibits the enzymatic activity of SrtA *in vitro*. (**A**) Effect of Stp1 and DTT on the enzyme activity of phosphorylated SrtA_ΔN24_ (P~SrtA_ΔN24_) *in vitro*. P~SrtA_ΔN24_ was purified from *E. coli*-expressed SrtA_ΔN24_ that has been phosphorylated by the cell lysates of Δ*stp1* strain. Stp1 and DTT were respectively added to the reaction mixture in order to dephosphorylate P~SrtA_ΔN24_. (**B**) Effect of AcP-treatment on the *in vitro* enzyme activity of SrtA_ΔN24_. Purified SrtA_ΔN24_ expressed in *E. coli* was treated by AcP (25 mM) as described in materials and methods. Stp1 was added to the reaction mixture in order to dephosphorylate the AcP-mediated phosphorylation of SrtA_ΔN24_. (**C**) Effect of Stk1_KD_ and Stp1 on the enzyme activity of SrtA_ΔN24_ *in vitro*. Recombinant Stk1_KD_ was added to the reaction mixture in order to phosphorylate SrtA_ΔN24_ while Stp1 was added in order to dephosphorylate SrtA_ΔN24_. In (A) to (C), a fluorescently self-quenched peptide, Abz-LPETG-Dnp, was used to mimic the surface protein substrate, which contains a LPETG sorting signal. When Abz-LPETG-Dnp is cleaved by SrtA, the fluorophore Abz group is released from the quencher Dnp group, and an enhanced fluorescence signal was detected. RFI, relative fluorescence intensity.

Cys184 is essential for the functions of SrtA [14], we thus reasoned that its phosphorylation may abolish the enzyme activity of SrtA. We performed a fluorescence resonance energy transfer (FRET) assay with phosphorylated SrtA_ΔN24_ (P~SrtA_ΔN24_) that has been phosphorylated by the cell lysates of the *stk1*^+++^ strain, where cysteine residue is the primary phosphorylation site (Figure 2C and 2D). In this assay, Stp1 and DTT were respectively added to a similar reaction in order to dephosphorylate the P~SrtA_ΔN24_. Our results showed that the treatment of purified P~SrtA_ΔN24_ with Stp1 obviously increased its ability to cleave the abz-LPETG-dnp substrate (Figure 4A). DTT-treatment also gave rise to an obvious increase of SrtA enzyme activity (Figure 4A). Thus, cysteine phosphorylation appears to inhibit the enzyme activity of SrtA. However, it was a little unexpected that the purified P~SrtA_ΔN24_ still exhibited enzyme activity (Figure 4A) and the reasons for this are currently unknown. It may be due to the chemical liability of the S-linked phosphorylation of SrtA and/or the fact that the purified protein sample is a mixture of phosphorylated SrtA_ΔN24_ with different phosphorylation events, except for the cysteine phosphorylation.

AcP – mediated phosphorylation also inhibited the enzyme activity of SrtA. As shown, AcP-treated SrtA_ΔN24_ displayed almost no enzyme activity in our FRET assay while retreatment of the AcP-treated SrtA_ΔN24_ with Stp1 obviously increased its ability to cleave the abz-LPETG-dnp substrate (Figure 4B). AcP might have a role in the regulation of SrtA-mediated anchoring of surface proteins in *S. aureus*. However, this hypothesis awaits further investigations. Nonetheless, these data clearly suggest that the inhibitory effect of AcP on the *in vitro* enzyme activity of SrtA is mediated at least in part by protein phosphorylation.

Additionally, we observed that the purified *E. coli*-expressed *S. aureus* SrtA_ΔN24_ can cleave the abz-LPETG-dnp substrate efficiently (Figure 4B andC) and as expected [12] while it failed to do this in the presence of Stk1_KD_ (Figure 4C). When Stp1 was subsequently added to the phosphorylation reaction mixture, the enzyme activity of SrtA_ΔN24_ was restored (Figure 4C), indicating that Stk1-mediated phosphorylation inhibits the enzyme activity of SrtA. Based on these data, we conclude that the enzyme activity of SrtA is regulated by phosphorylation, at least *in vitro*.

## Deletion of *stp1* in *S. aureus* Newman produces *srtA*-deficient phenotypes

**Figure 5.**
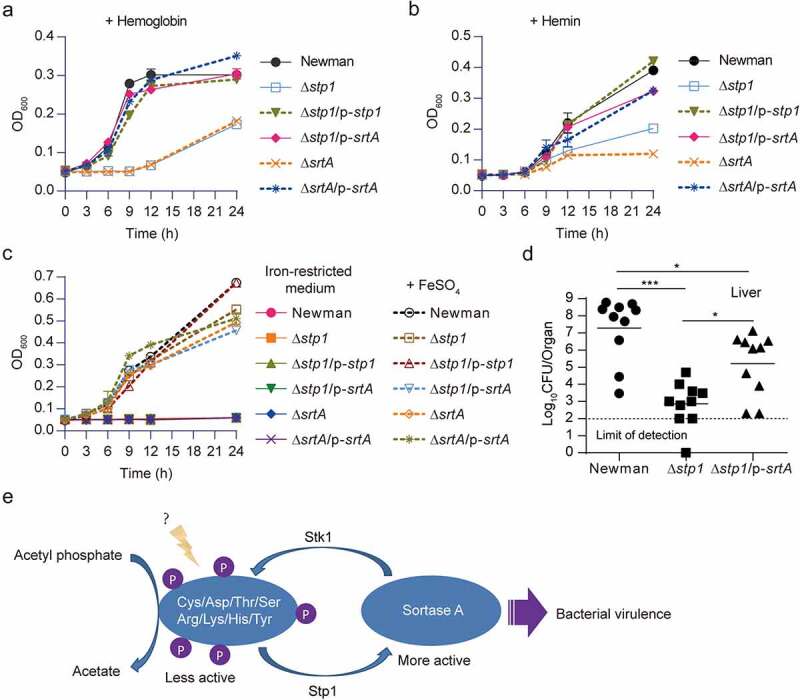
Overexpression of *srtA* alleviates the defects of *stp1* deletion mutant. (A and B) Effect of constitutive expression of either *stp1* or *srtA* on the bacterial growth of Δ*stp1* mutant in iron-restricted RPMI medium (Chelex 100 resin-treated RPMI) supplemented with either haemoglobin (2.5 μg/ml) or haem (2 μM) as the sole iron source. data shown represent the mean ± SD from triplicate experiments. (**C**) Growth curves for *S. aureus* in iron-restricted RPMI medium (Chelex 100 resin-treated RPMI) supplemented with or without FeSO_4_ (300 μM). Data shown represent the mean ± SD (*n* = three biological replicates). (**D**) Bacterial virulence of *S. aureus* Newman and its variants in mouse infection models. BALB/c female mice (*n* = 10) were retro-orbitally injected with 4 × 10^6^ CFU *S. aureus* bacteria; *S. aureus* colonization in murine liver was measured after 5 days of infection; each symbol represents the value for an individual mouse; horizontal bars indicate observation means and dashed lines mark limits of detection; *p<0.05, ***p<0.001 (Mann–Whitney Test, two-tailed). *S. aureus* WT Newman harbours vector pYJ335 (Newman); *stp1* deletion mutant harbours plasmid pYJ335 alone (Δ*stp1*), pYJ335:*stp1*(Δ*stp1*/p-*stp1*) or pYJ335:*srtA* (Δ*stp1*/p-*srtA*); *srtA* deletion mutant (Δ*srtA*) harbours plasmid pYJ335 alone (Δ*srtA*) or (Δ*srtA*/p-*srtA*). (**E**) A proposed mechanism of SrtA phosphorylation. SrtA may act as a mediator in a variety of signalling pathways in *S. aureus* through integrating diverse phosphorylation events.

Deletion of *stp1* caused an increase in the phosphorylation level of SrtA in *S. aureus* (Figure 1E), we thus reasoned that *stp1* deletion may cause an inhibition of the function of SrtA because phosphorylation inhibited the *in vitro* enzymatic activity of SrtA (Figure 4). Supporting this notion, we found that, like the *srtA* deletion mutant [11,14], Δ*stp1* strain exhibited severe defects in bacterial growth in CRPMI (an iron-restricted medium) supplemented with either haemoglobin (Figure 5A) or haem (Figure 5B) as the sole iron source. Δ*stp1* strain displayed slight growth defect only when FeSO_4_ was supplemented as the sole iron source, and as expected, all the testing *S. aureus* strains fail to grow in the iron-restricted CRPMI medium (Figure 5C). The introduction of plasmid p-*srtA* can alleviate the growth defects of Δ*stp1* strain in the CRPMI medium supplemented with either haemoglobin or haem the sole iron source (Figure 5A and 5B).

To examine the role of SrtA in Stp1-mediated signalling pathway during infections, we subjected the WT Newman, Δ*stp1*, and Δ*stp1*/p-*srtA* to a murine model of abscess formation and measured the bacterial survival in livers. As shown in (Figure 5D), the Δ*stp1* strain exhibited a significantly decreased colonization of liver compared to that of mice infected with the wild-type Newman (~4.5 log decrease in the CFU), whereas the introduction of plasmid p-*srtA* significantly increased the bacterial survival (~2.1 log increase in the CFU) (Figure 5D), indicating that ectopic expression of SrtA can suppress the virulence defect of Δ*stp1*. Taken together, these results suggest that SrtA appears to be involved in the Stp1/Stk1 signalling pathway of *S. aureus* (Figure 5E).

## Discussion

Here, we provide the first evidence that SrtA, an attractive anti-infective target for Gram-positive infections, is subject to phosphorylation that can inhibit its function (Figure 5E). Notably, the catalytic site residue of *S. aureus* SrtA [14], Cys184, is a primary phosphorylation site in a cellular context (Figure 2 B to 2D). Because Cys184 is essential for the enzyme activity and the function of SrtA [14], it is thus difficult or impossible to interpret experiments involving mutations at this site unequivocally. However, consistent with previous reports that covalently modify this cysteine thiol abolished SrtA activity [14], the enzymatic activity of P~SrtA that has been primarily phosphorylated at Cys184 is severely reduced when compared to that of Stp1-treated P~SrtA (Figure 4A). Moreover, the enzyme activity of P~SrtA was obviously increased in the presence of DTT (Figure 4A), which is in line with the observation that Cys-phosphorylation is DTT-labile (Figure 2C) [36]. *S. aureus* SrtA is highly resistant to oxidative inhibition and maintaining a high reduction potential of the Cys184 [51]. It has also been reported that DTT fails to increase the enzymatic activity of SrtA even at a concentration of up to 10 mM [52]. Given these, it is very likely that phosphorylation at Cys184 will introduce additional charge and conformational change, resulting in reduced enzyme activity of SrtA. We should, however, keep in mind that phosphorylation at other residue(s) may also contribute to the inhibition of the enzyme activity of SrtA (Figure 4B andC).

*S. aureus* SrtA protein contains multiple phosphorylatable amino acids (Figure 2A). Hence, in *S. aureus* the properties of SrtA (i.e. activity and specificity) may be shaped by different phosphorylation events, given that multisite phosphorylation is an important mechanism for fine-tuned regulation of protein function [53,54]. Meanwhile, phosphorylation occurring at various amino acid residues implies that SrtA may act as a mediator in a variety of signalling pathways in *S. aureus* through integrating diverse phosphorylation events. Indeed, we found that, at least *in vitro*, environmental conditions determine the phosphorylation events of SrtA (Figure 2,3), and that phosphorylation inhibited its enzyme activity (Figure 4). Unraveling these aspects of signal transduction mechanisms will open up the new horizon towards understanding of the regulation of surface display of staphylococcal proteins.

In this study, we used an established FRET-based assay to monitor the enzyme activity of SrtA *in vitro* [52,55,56]. Although FRET has been extensively applied for probing the enzyme activity of SrtA, it is associated with several limitations [17]. For example, as the assay depends on the measurements of fluorescence, the quencher product would probably interfere with the results due to the inner filter effect [17]. Additionally, the presence of the cleaved product NH_2_-G-Dnp would decrease the fluorescence of Abz-LPET-OH when its concentration above 20 µM [52]. Besides, the FRET-based assay can only provide information on the initial cleavage of LPXTG-containing peptides, but not the kinetic parameters associated with the transpeptidation step, since the fluorophore Abz group has already been departed from the Dnp quencher group after the completion of the first step [56,57].

In sum, to our best knowledge, this is the first report of the phosphorylation of a sortase enzyme in bacteria. Further studies on the detailed mechanism by which the phosphorylation events affect the enzyme activity of SrtA will provide new insights into how this protein is regulated *in vivo* and contribute to the development of potent SrtA inhibitors which aim to combat Gram-positive infections. Moreover, in this study, we determined that Stk1 and Stp1 play a role in the phosphorylation/de-phosphorylation of SrtA *in vitro* (Figure 1 A to C) and these events are expected to happen in *vivo* (Figure 1 E to H). Since phosphorylation events inhibit SrtA enzyme activity (Figure 4), the inhibition of the Stp1 phosphatase might be a future pharmaceutical tool to reduce the activity of SrtA in *S. aureus*.

## Materials and methods

### Ethics statement

The animal study protocols were reviewed and approved by the Institutional Animal Care and Use Committee (IACUC) of the Shanghai Public Health Clinical Center (Permit Number: 2013P201) and were performed in strict accordance with the Regulations for the Administration of Affairs Concerning Experimental Animals approved by the State Council of People’s Republic of China [11–14-]1988). The laboratory animal usage licence number is SYXK-HU-2010–0098, certificated by Shanghai Committee of Science and Technology. The animals were randomized but the investigators were not blinded to the experimental conditions.

### Construction of plasmids

To construct the plasmid for the constitutive expression of *srtA*, a ca. 700 bp DNA covering 39 bp of *srtA* upstream region, the *srtA* gene, and a 40 bp downstream of *srtA* was amplified from *S. aureus* Newman genomic DNA with primers srtA-F and srtA-R (Table S2), and then cloned into pYJ335. The construct with *srtA* in the same orientation as the tetracycline-inducible xyl/tetO promoter was confirmed by DNA sequencing, yielding plasmid pYJ335:*srtA*.

To construct pYJ335:*6his-srtA*_ΔN59_, a DNA fragment was amplified from pET28a:*6his-srtA*_*ΔN59*_ with primers T7 promoter and T7 terminator (Table S2), and cloned into pYJ335. Primer pairs of pYJ335-F (Table S2) and T7 terminator were used to select the plasmid clones in which the genes are located downstream of the tetracycline-inducible *xyl*/*tetO* promoter.

To construct pET28a:*6his-stk1*_*KD*,_ DNA fragment encoding the Stk1 kinase domain (Stk1_KD_) (residues 1–291) of *S. aureus* strain Newman was amplified by PCR with primers stk1-KD-F (with *Eco*RI site) and stk1-KD-R (with *Xho*I site). After digestion with *Eco*RI and *Xho*I, the PCR product was inserted into the pET28a vector and yielded pET28a:*6his-stk1*_*KD*_.

To construct pET28a:*srtA*_*ΔN59*_, primer srtA_ΔN59_-F and srtA_ΔN59_-R (Table S2) were used. To introduce a single amino acid substitution (Cys to Ala substitution at C184) to *srtA* gene, primer pair srtA_C184A_-F/srtA_C184A_-R and plasmid DNA of either pET28a:*srtA*_*ΔN24*_ or pET28a:*srtA*_*ΔN59*_ were used as template. All the selected plasmid clones were sequenced to confirm that no additional mutations were introduced by PCR reactions.

### Construction of S. aureus ΔsrtA mutant

The gene replacement vector pKOR1 was used to construct an in-frame, unmarked *srtA* deletion mutant (Δ*srtA*). PCRs were performed to amplify flanking sequences of the intended deletion. The upstream fragment was amplified from *S. aureus* strain Newman genomic DNA using primers srtA-delete-up-F and srtA-delete-up-R (with *Eco*RI site), and the downstream fragment was amplified with primers srtA-delete-down-F (with *Eco*RI site) and srtA-delete-down-R. PCR products were digested with *Eco*RI, mixed together and ligated by T4 DNA ligase (New England Biolabs). The ligation product was amplified with primers srtA-delete-up-F and srtA-delete-down-R. Next, the PCR product was used for recombination with pKOR1, and the product was introduced to *E. coli* DH5α. The construction was sequenced to ensure that no additional mutations resulted. The resulting plasmid, pKOR1:Δ*srtA*, was electroporated to *S. aureus* RN4220, and subsequently into *S. aureus* Newman. The allelic replacement was performed as described previously [36] and the deletion of *srtA* was further confirmed by PCR and DNA sequencing.

### Recombinant protein purification from E. coli

Transformed bacteria were sub-cultured into 1000 ml LB medium supplemented with 50 μg/ml kanamycin at 37°C to an optical density of 0.5 at 600 nm. After lowering the temperature to 16°C (for Stp1 and SrtA expression) or 20°C (for Stk1_KD_ expression), 1 mM isopropyl-thiogalactopyranoside (IPTG) was added in order to induce the expression of protein and the bacteria were harvested by centrifugation after an overnight of shaking (250 rpm of aeration).

For the purification of 6His-Stk1_KD_, the pellets were resuspended in 30 ml buffer A [20 mM HEPES, pH 7.4; 150 mM NaCl, 10 mM imidazole, and 1 mM phenylmethanesulfonyl fluoride (PMSF)] and sonicated. The lysate was centrifuged at 15,000 g for 30 min, and the supernatant was loaded onto a HisTrap HP 5 ml column (GE Healthcare), which was then equilibrated with buffer A. The 6His-Stk1_KD_ was eluted with a linear gradient of 10–500 mM imidazole with buffer B [20 mM HEPES, pH 7.4; 150 mM NaCl, 500 mM imidazole, and 1 mM phenylmethanesulfonyl fluoride (PMSF)]. Fractions enriched for 6His-Stk1_KD_ were pooled and concentrated. The proteins were further purified by gel filtration (Superdex 75, GE Healthcare) with buffer C [20 mM HEPES, pH 7.4; 150 mM NaCl], stored at −80°C at a concentration of approximately 1 mg/ml in buffer D [10 mM HEPES, pH 7.4; 75 mM NaCl, 30% (wt/vol) glycerol].

Purification of Stp1 protein was performed as previously described [36]. For the purification of 6His-SrtA and its variants, buffer I [50 mM Tris-HCl, pH 7.5; 200 mM NaCl, 5 mM 2-mercaptoethanol, 40 mM imidazole] and buffer J [50 mM Tris-HCl, pH 7.5; 200 mM NaCl, 5 mM 2-mercaptoethanol, 300 mM imidazole] were used, and buffer K [20 mM Tris-HCl, pH 7.5, 50 mM NaCl] was used as gel filtration buffer. The purified proteins were stored in buffer L [10 mM Tris-HCl, pH 7.5, 25 mM NaCl, 30% (wt/vol) glycerol] at −80°C at a concentration of approximately 1 mg/ml until use.

### Protein phosphorylation by purified 6his-Stk1_KD_

For the detection of phosphoproteins with autoradiography, *in vitro* kinase assay were performed by incubating 0.5 μg 6His-Stk1_KD_ without or with 4 μg of the desired proteins (6His-SrtA_ΔN24_ or 6His-SrtA_ΔN24 C184A_, as indicated) in phosphorylation reaction buffer [50 mM Tris-HCl, pH 7.5; 0.1 mM EDTA, 1 mM MnCl_2_, 5 mM MgCl_2_] in the presence of 10 μCi [γ-^32^P]ATP at room temperature for 30 min. Likewise, for dephosphorylation assay, reaction was incubated with 0.5 μg Stp1 for an additional 30 min at room temperature. Reactions were performed in volumes of 20 μl, and an equal volume of protein storage buffer was added as needed in order to maintain a constant reaction condition. Reactions were stopped by the addition of 5 μl 5 × SDS loading buffer (without DTT), and the samples were resolved on a 12% SDS-PAGE, visualized by autoradiography.

Similarly, for the detection of phosphoproteins by Pro-Q Diamond stain, the *in vitro* kinase assay was performed by incubating 2 μg 6His-Stk1_KD_ without or with 4 μg of the desired proteins in phosphorylation reaction buffer supplemented with 1 mM ATP at room temperature for 30 min. Likewise, the dephosphorylation assay was performed by incubating the reactions with 1 μg Stp1 for an additional 30 min at room temperature. Reaction was stopped by the addition of 5 μl 5 × SDS loading buffer (with or without DTT, as indicated). Samples were resolved on a 12% SDS-PAGE. Following electrophoresis, proteins were immersed 30 min in fixing solution [50% methanol, 10% acetic acid]. The gel was subsequently washed in distilled water and treated for 90 min with Pro-Q Diamond Phosphoprotein Gel Stain. After destaining with destain buffer [20% acetonitrile, 50 mM sodium acetate, pH 4.0], phosphorylated species were visualized by using Tanon-5200 multi.

All the gels were subsequently stained with Coomassie blue to ensure the quantity of the loaded proteins.

### Protein phosphorylation by bacterial cell lysates

*S. aureus* overnight cultures were diluted 100-fold in fresh TSB medium, and grown in a 250-ml flask with a flask volume to medium volume ratio of 5:1, shaking with 250 rpm at 37°C for 6 h. 10 ml cultures were harvested by centrifugation, washed once and suspended in 1 ml of phosphorylation buffer [50 mM Tris-HCl, pH 7.5; 0.1 mM EDTA, 1 mM MnCl_2_, 5 mM MgCl_2_] supplied with 1 μl protease inhibitor cocktail (Thermo) and 1 μl phosphatase inhibitor cocktail 2 (Sigma). The mixture was homogenized by mechanical disruption (Fast Prep FP2400 instrument; Qbiogene) and then removed debris by centrifuging and filtrating through 0.45  μm filter.

For phosphorylation assay, 30 μg cell lysates were incubated with or without the desired 6His-proteins (~2 μg) in a phosphorylation buffer in the presence of 10 μCi of [γ-^32^P] ATP. Reactions were performed in volumes of 20 μl at room temperature for 30 min, and stopped by adding 5 μl 5 × SDS loading buffer (with or without DTT, as indicated). The phosphorylation signal was visualized by autoradiography and the gel was subsequently stained with Coomassie blue to ensure the quantity of the loaded proteins.

### Protein phosphorylation by acetyl phosphate

Recombinant SrtA proteins (~4 µg) purified from *E. coli* were incubated with or without lithium potassium acetyl phosphate (AcP) (a final concentration of 25 mM) in a phosphorylation reaction buffer. Reactions were performed in volumes of 20 μl at 37°C for 1 h. 6his-tagged *Pseudomonas aeruginosa* ClpP protein (6His-ClpP) [58], which contains 13 aspartic acid residues, was included as a recombinant protein controls, where appropriate. Reactions were stopped by adding 5 μl 5 × SDS loading buffer (with 100 mM DTT), and the samples were resolved on SDS-PAGE, and visualized by Pro-Q Diamond stain, subsequently stained with Coomassie blue to ensure the quantity of the loaded proteins. The signal intensity was determined by densitometric analysis using ImageQuant software.

For the dephosphorylation assay, recombinant proteins that have treated by AcP were loaded to a desalting spin column (BIORAD Bio-Spin® 6 column) for buffer exchange with 10 mM Tris buffer (10 mM Tris-HCl, pH 7.4) containing 0.02% sodium azide. After that, AcP-treated proteins (~4 µg) were incubated with or without Stp1 (~1 µg) at 37°C for 30 min, or subject to heating (95°C for 10 min). An equal volume of protein storage buffer was added as needed in order to maintain a constant reaction condition and reactions were performed in a phosphorylation reaction buffer in volumes of 20 μl.

### Dephosphorylation of 6his-SrtA_ΔN59_ purified from S. aureus

Overnight culture of *S. aureus* was diluted 1:100 in fresh 1,000 ml TSB (containing 10 μg/ml chloramphenicol, 10 μg/ml erythromycin, and 0.1 μg/ml anhydrotetracycline) and inculcated at 37°C with for 6 h with shaking (250 rpm of aeration). Cultures were harvested by centrifugation, washed once and suspended in 40 ml of buffer M [20 mM Na_3_PO_4_, pH 7.5; 200 mM NaCl] supplied with 4 μl protease inhibitor cocktail and 4 μl phosphatase inhibitor cocktail 2. The mixture was homogenized by mechanical disruption (Fast Prep FP2400 instrument; Qbiogene) and then removed debris by centrifuging and filtrating through 0.45 μm filter. 6His-SrtA_ΔN59_ was enriched with Ni-NTA beads and eluted with buffer N [20 mM Na_3_PO_4_, pH 7.5; 200 mM NaCl, 300 mM imidazole]. The eluted proteins were concentrated to about 1 mg/ml using Amicon Ultra centrifugal filter.

For protein dephosphorylation, 6His-SrtA_ΔN59_ proteins (~4 μg) were incubated with Stp1 (~1 μg) or DTT in a phosphorylation buffer for final volume of 20 μl. In the control reaction (6His-SrtA_ΔN59_ alone), an equal volume of protein storage buffer for Stp1 was added. The reaction mixtures were maintained at room temperature for 30 min and then were stopped by adding 5 μl 5 × SDS loading buffer (without DTT). The samples were resolved on SDS-PAGE, and visualized by Pro-Q Diamond stain, subsequently stained with Coomassie blue to ensure the quantity of the loaded proteins. The signal intensity was determined by densitometric analysis using ImageQuant software.

### LC-MS/MS identification of phosphorylation sites on SrtA

6His-SrtA_ΔN59_ (~60 μg) purified from *E. coli* was added into cell free extracts (~300 µg) of *stk1*^+++^ (Newman/pYJ335:*stk1*) and incubated with 1 mM ATP for 30 min at room temperature. The trypsin digested sample was analysed using an LTQ-Orbitrap Elite or Q exactive mass spectrometer using a nanospray source. After phosphopeptide enrichment by Fe-NTA spin column, 50 µg sample was loaded onto the MudPIT column. Full MS spectra (from m/z 300–1800) were acquired by the precursor ion scan using the Orbitrap analyser with resolution *R* = 60, 000 at m/z 400, followed by 20 MS/MS events. The twenty most intense ions were sequentially isolated and subjected to CID or HCD for fragmentation.

Protein identification analysis was done with Integrated Proteomics Pipeline (IP2, Integrated Proteomics Applications, Inc. San Diego, CA. http://www.integratedproteomics.com) using ProLuCID/Sequest, DTASelect2. Tandem mass spectra were extracted into ms2 files from raw files using RawExtract 1.9.9 (http://fields.scripps.edu/downloads.php) and were searched against Uniprot *Staphylococcus aureus* Newman local database. Database search criteria are as follows: mass tolerances for precursor ions were set at 12 ppm and for MS/MS were set at 600 ppm. Cysteine, serine, threonine, tyrosine, histidine, arginine, lysine and aspartic phosphorylation were considered as variable modifications and we require one peptide per protein and at least one tryptic terminus for each peptide identification.

## Co-immunoprecipitation

Overnight cultures of *S. aureus* were diluted 1:100 and grown in TSB in a 250-ml flask with a tube volume-to-medium volume ratio of 5:1, shaking with 250 rpm at 37°C for 6 h. 10 ml cultures were harvested by centrifugation, washed once and suspended in 1 ml of lysis buffer provided in the CO-IP kit (Pierce) supplied with 1 μl protease inhibitor cocktail (Thermo). The mixture was homogenized by mechanical disruption (Fast Prep FP2400 instrument; Qbiogene) and then the debris was removed by centrifuging and filtrating through 0.45 μm filters. 500 µl clarified lysates were incubated with the resins which immobilized with specific antibodies or not for 12 h at 4°C. The bound materials were washed and eluted following the manufacturer’s recommendation. Total samples (clarified lysates) and the immunocomplexes eluted from the resins were analysed by western blot with specific antibodies.

## Western blot analysis

15 μl of 5 × SDS loading buffer [250 mM Tris-HCl, pH 6.8; 10% SDS; 0.5% bromophenol blue; 50% glycerol; 100 mM DTT] was added into 60 μl eluted immunocomplexes and then heated at 100°C for 10 min. SDS-PAGE was carried out subsequently. Thereafter, proteins were transferred to a PVDF (Bio-Rad) membrane at 350 mA for 60 min. The membrane was incubated with the primary antibody in 10 ml of 5% (wt/vol) skim milk at 4°C overnight following the blocking step [10 ml of 5% (wt/vol) skim milk] at room temperature for 2 h, and then washed three times at room temperature for 30 min in TBST buffer [10 mM Tris-HCl, pH 7.5, 150 mM NaCl, and 0.04% Tween 20]. Then, membranes were incubated with the secondary antibody for 2 h at room temperature and washed three times for 30 min in TBST. The chemiluminescent detection reaction was performed and detected by Tanon-5200 multi. The following primary antibodies were used: anti-Sortase A antibody (Abcam, #ab13959) with a 1:3000 dilution, anti-Stk1 polyclonal antibody (prepared by immunizing rabbits with the purified 6His-Stk1_KD_, Shanghai Immune Biotech CO., Ltd) with a 1:1000 dilution, anti-Stp1 polyclonal antibody (prepared by immunizing rabbits with the purified Stp1 protein, Shanghai Immune Biotech CO., Ltd) with a 1:500 dilution. For detection of the primary antibodies, anti-rabbit IgG (GE Healthcare, #NA934) with a 1:5000 dilution was used.

## Enrichment of phosphorylated SrtA

6His-SrtA_ΔN24_ proteins (~15 mg) purified from *E. coli* was incubated with the cell lysates (~75 mg) of *S. aureus* Δ*stp1* strain in the presence of 1 mM ATP in phosphorylation buffer. Reactions were performed in volumes of 40 ml at room temperature for 30 min. 6His-SrtA_ΔN24_ was purified with HisTrap HP 5 ml column and the phosphoprotein purification kit (Qiagen) was used to further enrichment of the phosphorylated forms of 6His-SrtA_ΔN24_ (P ~ 6His-SrtA_ΔN24_) following the manufacturer’s instruction. The enriched protein was concentrated to 1 mg/ml in phosphoprotein elution buffer [2.92 g NaCl, 7.42 g K_2_HPO_4_, 1.15 g KH_2_PO_4_, pH 7.5, ddH_2_O to 1,000 ml].

## Enzymatic activity of SrtA

A FRET assay was used to monitor the *in vitro* activity of SrtA as described previously using LPETG fluorescent peptides (abz-LPETG-dnp) as a substrate [52]. In a typical FRET assay, a 100 μl reaction mixture contained 3 μM LPETG fluorescent peptides (abz-LPETG-dnp), 4 μg 6His-SrtA_ΔN24_ in assay buffer [50 mM Tris-HCl, pH 7.5; 150 mM NaCl, 5 mM CaCl_2_]. An equal volume of protein storage buffer (and/or phosphorylation buffer) was added as needed in order to maintain constant reaction conditions.

Briefly, for the dephosphorylation of 6His-SrtA_ΔN24_ that has been phosphorylated by the cell lysates of *S. aureus* Δ*stp1*, 12 µg phosphorylated proteins were incubated with either Stp1 (~3 µg) or DTT (final concentration of 1 mM) at 37°C for 30 min in phosphorylation buffer in a total volume of 60 µl and after that, 20 μl sample mixture is used for subsequent FRET assay. For the dephosphorylation of 6His-SrtA_ΔN24_ that has been phosphorylated by AcP, AcP-terated 6His-SrtA_ΔN24_ (12 µg) was incubated with 3 µg Stp1 in phosphorylation buffer in a total volume of 60 µl and incubated at 37°C for 30 min, and 20 μl sample mixture is then used for subsequent FRET assay.

For the phosphorylation of 6His-SrtA_ΔN24_ by Stk1, *in vitro* kinase assay was performed in 60 μl of phosphorylation buffer containing 6His-SrtA_ΔN24_ (~12 μg) and 6His-Stk1_KD_ (~6 μg) in the presence of 1 mM ATP at 37°C for 30 min. Likewise, for the dephosphorylation assay, reaction was incubated with 3 μg Stp1 for an additional 30 min at room temperature. 20 μl sample mixture is then used for subsequent FRET assay, respectively.

Reaction sample was distributed in 96-well black plates (PerkinElmer), and the increase in fluorescence intensity was monitored every 1 min for 40 min using Synergy 2 (Biotek) plate reader at an excitation wavelength of 360 nm and emission wavelength of 460 nm. Triplicate measurements were taken for each sample, and the data are reported as mean.

## Bacterial growth curves

The *S. aureus* cultures were grown overnight in RPMI supplied with 1% (wt/vol) casamino acids and 500 μM 2,2-dipyridyl (DIP) to generate iron-restricted condition. The cells were washed twice in CRPMI (Chelex treated RPMI) containing 500 μM DIP, and normalized to OD_600_ to 5.0. Subcultured 10 μl bacteria suspension into 1 ml CRPMI containing 25 μM ZnCl_2_, 25 μM MnCl_2_, 1 mM MgCl_2_, 100 μM CaCl_2_, 0.5 mM DIP, and grown in 15 ml screw-cap conical tube at 37°C with aeration. 2.5 μg/ml (wt/vol) haemoglobin (TCI, H1293), 2 μM haem (Alfa Aesar, A18518) or 300 μM FeSO_4_ was supplemented (at a final concentration) as indicated and OD_600_ was monitored in every 3 h or 12 h by removed 50 μl culture mixed with 150 μl PBS in 96-well plates. The data are reported as mean ± SD.

## Mouse infection models

*S. aureus* overnight cultures were diluted 1:100 and grown in fresh TSB in a 250-ml flask with a tube volume-to-medium volume ratio of 5:1, shaking with 250 rpm at 37°C for 3 h. Bacteria were harvested and washed twice with ice-cold PBS. The CFU per millilitre was determined before mice were inoculated. Mouse infection models were carried out as described previously [7,59]. 6 to 8-week-old female BALB/c mice were obtained from SIPPR-BK Lab Animal ltd (http://www.slarc.org.cn) and housed under specified pathogen-free conditions.

Mice were anaesthetized with pentobarbital sodium (intra-peritoneal injection, 80 mg/kg) and were challenged with either 4 × 10^6^ CFU of *S. aureus* Newman and its variants. Animals were euthanized 5 days post infection. Livers were aseptically removed and homogenized in PBS in order to obtain single-cell suspensions, and serial dilutions of each organ were plated on TSA plates for the enumeration of CFU. In this experiment, mice were also received intra-peritoneal injections every 24 h with a total dose of 40 mg/kg erythromycin hydrochloride (in order to maintain the plasmid) and 10 μg/kg anhydrotetracycline (in order to induce *srtA* expression) dissolved in sterile double-distilled water (ddH_2_O) for 120 h (5 d). The statistical significance was determined by the Mann–Whitney Test (two-tailed).

## Supplementary Material

Supplemental Material

## Data Availability

The authors confirm that the data supporting the findings of this study are available within the article and its supplementary materials.
