# Supplementary material for: The enzyme activity of sortase A is regulated by phosphorylation in Staphylococcus aureus
Source: Virulence. 2023 Feb 10;14(1):2171641. doi: 10.1080/21505594.2023.2171641 (PMC9928477; doi:10.1080/21505594.2023.2171641)
Supplement: Supplemental Material [file KVIR_A_2171641_SM6840.docx]

**Supporting Information**

**Phosphorylation of sortase A in *Staphylococcus aureus***

Feifei Chen^1,2#^, Hongxia Di^2,3#^, Yanhui Wang^2,3^, Chao Peng^4^, Rongrong Chen^3,5^, Huiwen Pan^3,5^, Cai-guang Yang^2,3,5^, Haihua Liang^1,6*^, Lefu Lan^1,2,3,5*^

^1^College of Life Science, Northwest University, Xi'an 710127, China.

^2^State Key Laboratory of Drug Research, Shanghai Institute of Materia Medica, Chinese Academy of Sciences, Shanghai 201203, China;

^3^University of Chinese Academy of Sciences, Beijing 100049, China

^4^National Facility for Protein Science in Shanghai, Zhangjiang Lab, Shanghai Advanced Research Institute, Chinese Academy of Science, 201210, Shanghai, China.

^5^Hangzhou Institute for Advanced Study, University of Chinese Academy of Sciences, Hangzhou 310024, China

^6^School of Medicine, Southern University of Science and Technology, Shenzhen, China.

^#^F.C. and H.D. contributed equally to this work.

^*^To whom correspondence should be addressed, Email: [llan@simm.ac.cn](mailto:llan@simm.ac.cn) (L.L.) or lianghh@sustech.edu.cn

The authors declare no conflict of interest.

Running Title: Phosphorylation of SrtA

**Table S1. Plasmids and strains used in this study**

| Strains or plasmids | Relevant genotype or characteristic | Source |
| --- | --- | --- |
| Plasmids | | |
| pKOR1 | Gene replacement vector for *S. aureus* gene, Amp^r^, Cm^r^ | ([1](#_ENREF_1)) |
| pYJ335 | *E. coli*-*S. aureus* shuttle vector, Cm^r^, Erm^r^ | ([2](#_ENREF_2)) |
| pET28a | Km^r^, protein expression vector | Novagen |
| pKOR1::Δ*srtA* | pKOR1 derivative, for deletion of *srtA* gene | This study |
| pYJ335::*srtA* | p-*srtA*; pYJ335 derivative carrying *srtA* gene of *S. aureus* Newman in the downstream of the *xyl*/*tetO* promoter | This study |
| pYJ335::*stk1* | pYJ335 derivative carrying *stk1* gene of *S. aureus* Newman in the downstream of the *xyl*/*tetO* promoter | ([3](#_ENREF_3)) |
| pYJ335::*stp1* | p*-stp1*; pYJ335 derivative carrying *stp1* gene of *S. aureus* Newman in the downstream of the *xyl*/*tetO* promoter | ([3](#_ENREF_3)) |
| pYJ335::*6his*-*srtA_ΔN59_* | pYJ335 derivative carrying *6his-srtA* gene in the downstream of the *xyl*/*tetO* promoter, deleted the N-terminal 59 residues encompassing the membrane anchor sequence of SrtA | This study |
| pMCSG19::*stp1* | pMCSG19 derivative carrying *stp1* | ([3](#_ENREF_3)) |
| pET28a::*stk1_KD_* | pET28a derivative carrying *stk1* kinase domain of *S. aureus* Newman | This study |
| pET28a::*srtA_ΔN24_* | pET28a derivative carrying *srtA* gene of *S. aureus* Newman, deleted the N-terminal 24 residues encompassing the membrane anchor sequence of SrtA | ([4](#_ENREF_4)) |
| pET28a::*srtA_ΔN24 C184A_* | pET28a::*srtA_ΔN24_* carrying alanine substitution mutant at the site cysteine 184 of SrtA_ΔN24_ | This study |
| pET28a::*srtA_ΔN59_* | pET28a derivative carrying *srtA* gene of *S. aureus* Newman, deleted the N-terminal 59 residues encompassing the membrane anchor sequence of SrtA | This study |
| pET28a:: *srtA*_ΔN59_ *_C184A_* | pET28a::*srtA_ΔN59_* carrying alanine substitution mutant at the site cysteine 184 of SrtA_ΔN59_ | This study |
| *Staphylococcus aureus* | | |
| Newman | Wild-type, *S. aureus* ATCC 25904 | ([5](#_ENREF_5)) |
| RN4220 | Derivative of 8325-4 that accepts plasmids | ([6](#_ENREF_6)) |
| Δ*srtA* | *srtA* gene deletion mutant of Newman strain | This study |
| Δ*stp1* | *stp1* gene deletion mutant of Newman strain | ([3](#_ENREF_3)) |
| *stk1-*I | A transposon insertion in the *stk1* gene of Newman strain | ([3](#_ENREF_3), [7](#_ENREF_7)) |
| Newman/pYJ335::*stk1* | *stk1*^+++^; wild-type *S. aureus* Newman carrying plasmid pYJ335::*stk1* | This study |
| Newman/pYJ335 | Wild-type *S. aureus* Newman carrying plasmid pYJ335 | This study |
| Δ*stp1*/pYJ335 | Newman *stp1* deletion mutant carrying plasmid pYJ335 | This study |
| Δ*stp1*/pYJ335::*stp1* | Newman *stp1* deletion mutant carrying plasmid pYJ335::*stp1* | This study |
| Δ*stp1*/pYJ335::*srtA* | Δ*stp1*/p-*srtA*; Newman *stp1* deletion mutant carrying pYJ335::*srtA* | This study |
| Newman/pYJ335::*6his*-*srtA_ΔN59_* | Wild-type *S. aureus* Nemwan carrying plasmid pYJ335::*6his*-*srtA_ΔN59_* | This study |
| Δ*stp1*/pYJ335::*6his*-*srtA_ΔN59_* | Newman *stp1* mutant carrying plasmid pYJ335::*6his*-*srtA*_ΔN59_ | This study |
| *E. coli* | | |
| BL21(DE3) | F^−^ *ompT hsdS_B_* (r_B_^−^ m_B_^−^) *gal dcm met* (DE3) | Laboratory stock |
| DH5α | *endA hsdR17 supE44 thi-1 recA1 gyrA relA1*Δ(*lacZYA-argF*)*U169 deoR* (*φ80dlac*Δ(*lacZ*)*M15*) | Laboratory stock |

Amp^r^, ampicillin resistance; Kan^r^, kanamycin resistance; Cm^r^, chloroamphenicol resistance; Erm^r^, erythromycin resistance; Tc^r^, tetracycline resistance

**Table S2. Primers used in this study**

| Primers | Sequence (5′to 3′) |
| --- | --- |
| srtA-delete-up-F | GGGGACAAGTTTGTACAAAAAAGCAGGCT TTGATAAAAAAGAAGCATCTGTC |
| srtA-delete-up-R | AACGAATTCCAAACAAATATGCTGCCACTA |
| srtA-delete-down-F | AACGAATTCATCTATTACGCTAATGGATGAAT |
| srtA-delete-down-R | GGGGACCACTTTGTACAAGAAAGCTGGGTCTTGGTCAGATCAATCACATT |
| srtA-F | TAACAGGCATTGTGAAATGT |
| srtA-R | CTGTTTTCCACTCAATATATTC |
| stk1-KD-F | AACGAATTCATGATAGGTAAAATAATAAATGAA |
| stk1-KD-R | AAACTCGAGTACCGCTATCGTTTTCATTT |
| srtA_ΔN59_-F | AAACATATGCAAGCTAAACCTCAAATTCC |
| srtA_ΔN59_-R | CCGCTCGAGTTATTTGACTTCTGTAGCTACAA |
| srtA_C184A_-F | AACAATTAACATTAATTACTGCTGATGATTACAATGAAAA |
| srtA_C184A_-R | GTCTTTTCATTGTAATCATCCGAAGTAATTAATGTTAATT |
| pYJ335-F | CAATACAATGTAGGCTGCTCTACAC |
| T7 promoter | AAATAATACGACTCACTATAGG |
| T7 terminator | ATGCTAGTTATTGCTCAGCGG |

**Table S3. Detected phosphopeptides and phosphorylation sites of SrtA by LC-MS/MS**

| Sequence | Position | Phosphorylation Sites | MH+(obs.) | MH+(cal.) | Error  (ppm) |
| --- | --- | --- | --- | --- | --- |
| K.PHIDNY(79.9663)LHDKDKDEK.I | 26-42 | Y32 | 1948.8589 | 1946.8644 | -6.3 |
| K.PHIDNYLHDKDK(79.9663)DEK.I | 26-42 | K38 | 1947.857 | 1946.8644 | -5.5 |
| K.QQAK(79.9663)PQIPKDKSKVAGYIEIPDADIKEPVYPGPATPEQLNR.G | 58-100 | K62 | 4611.339 | 4609.37 | -8.2 |
| K.QQAKPQIPKDKSKVAGY(79.9663)IEIPDADIKEPVYPGPATPEQLNR.G | 58-100 | Y75 | 4610.4272 | 4609.37 | 11.7 |
| K.PQIPKDKS(79.9663)KVAGY(79.9663)IEIPDADIKEPVYPGPATPEQLNR.G | 62-100 | S70 Y75 | 4237.131 | 4234.087 | 8 |
| K.PQIPK(79.9663)D(79.9663)KS(79.9663)K(79.9663)VAGY(79.9663)IEIPDADIKEPVYPGPATPEQLNR.G | 62-100 | K67 D68 S70  K71 Y75 | 4476.9556 | 4473.986 | -9 |
| K.PQIPKD(79.9663)KSK(79.9663)VAGYIEIPDADIKEPVYPGPATPEQLNR.G | 62-100 | D68 K71 | 4237.131 | 4234.087 | 8 |
| K.DKS(79.9663)KVAGYIEIPDADIKEPVYPGPATPEQLNR.G | 67-100 | S70 | 3590.7798 | 3590.7778 | 0.5 |
| K.DKSK(79.9663)VAGYIEIPDADIKEPVYPGPATPEQLNR.G | 67-100 | K71 | 3590.7798 | 3590.7778 | 0.5 |
| K.VAGY(79.9663)IEIPDADIKEPVYPGPATPEQLNR.G | 71-100 | Y75 | 3134.5195 | 3132.5288 | -5.1 |
| K.VAGY(79.9663)IEIPDADIKEPVYPGPATPEQLNR.G | 71-100 | Y75 | 3134.5227 | 3132.5288 | -4.1 |
| K.VAGYIEIPD(79.9663)ADIKEPVYPGPATPEQLNR.G | 71-100 | D80 | 3133.519 | 3132.5288 | -4.2 |
| K.VAGYIEIPDAD(79.9663)IKEPVYPGPATPEQLNR.G | 71-100 | D82 | 3134.51 | 3132.5288 | -8.1 |
| R.GVS(79.9663)FAEENESLDDQNISIAGHTFIDRPNYQFTNLK.A | 99-135 | S102 | 4050.8352 | 4049.8552 | -5.8 |
| R.GVS(79.9663)FAEENESLDDQNISIAGHTFIDRPNYQFTNLK.A | 99-135 | S102 | 4051.8474 | 4049.8552 | -3.6 |
| R.GVSFAEENES(79.9663)LDDQNISIAGHTFIDRPNYQFTNLK.A | 99-135 | S109 | 4051.853 | 4049.8552 | -2.2 |
| R.GVSFAEENES(79.9663)LDDQNISIAGHTFIDRPNYQFTNLK.A | 99-135 | S109 | 4050.8386 | 4049.8552 | -4.9 |
| R.GVSFAEENESLD(79.9663)DQNISIAGHTFIDRPNYQFTNLK.A | 99-135 | D111 | 4051.8613 | 4049.8552 | -0.1 |
| R.GVSFAEENESLDD(79.9663)QNISIAGHTFIDRPNYQFTNLK.A | 99-135 | D112 | 4050.8555 | 4049.8552 | -0.8 |
| R.GVSFAEENESLDDQNIS(79.9663)IAGHTFIDRPNYQFTNLK.A | 99-135 | S116 | 4050.854 | 4049.8552 | -1.1 |
| R.GVSFAEENESLDDQNIS(79.9663)IAGHTFIDRPNYQFTNLK.A | 99-135 | S116 | 4050.8418 | 4049.8552 | -4.1 |
| R.GVSFAEENESLDDQNISIAGH(79.9663)TFIDRPNYQFTNLK.A | 99-135 | H120 | 4051.8545 | 4049.8552 | -1.8 |
| R.GVSFAEENESLDDQNISIAGHT(79.9663)FIDRPNYQFTNLK.A | 99-135 | T121 | 4051.8567 | 4049.8552 | -1.3 |
| R.K(79.9663)Y(79.9663)K(79.9663)MT(79.9663)S(79.9663)IR(79.9663)DVKPTDVGVLDEQK.G | 151-172 | K152 Y153 K154  T156 S157 R159 | 3032.1807 | 3030.1572 | 5.5 |
| K.MT(79.9663)SIRDVKPTDVGVLDEQK.G | 154-174 | T156 | 2212.072 | 2211.0725 | -1.7 |
| K.MTS(79.9663)IRDVKPTDVGVLDEQK.G | 154-174 | S157 | 2212.0742 | 2211.0725 | -0.7 |
| K.MTSIR(79.9663)DVKPTDVGVLDEQK.G | 154-174 | R159 | 2212.0742 | 2211.0725 | -0.7 |
| R.D(79.9663)VKPTDVGVLDEQK.G | 159-174 | D160 | 1623.7599 | 1622.7672 | -6.6 |
| R.DVK(79.9663)PTDVGVLDEQK.G | 159-174 | K162 | 1623.7544 | 1622.7672 | -10 |
| R.DVKPT(79.9663)DVGVLDEQK.G | 159-174 | T164 | 1622.7651 | 1622.7672 | -1.3 |
| R.DVKPT(79.9663)DVGVLDEQK.G | 159-174 | T164 | 1623.7657 | 1622.7672 | -3 |
| R.DVKPT(79.9663)DVGVLDEQKGK.D | 159-176 | T164 | 1808.8829 | 1807.8837 | -2.3 |
| K.DKQLT(79.9663)LITCDDYNEK.T | 175-191 | T180 | 1935.8575 | 1935.8405 | 8.8 |
| K.QLTLITC(79.9663)DDYNEK.T | 177-191 | C184 | 1635.7148 | 1635.6971 | 10.8 |


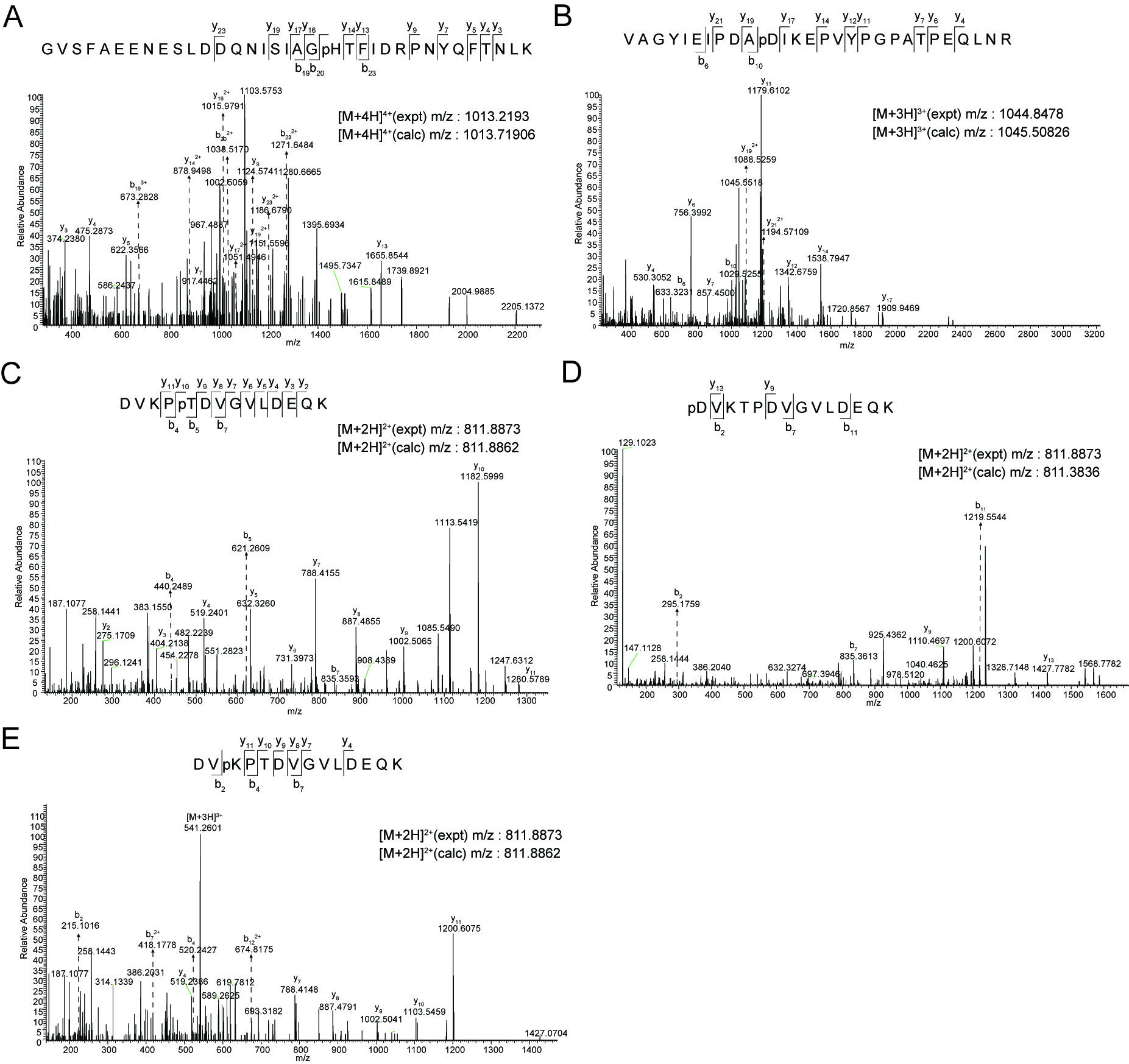


**Figure S1. The LC-MS/MS spectrums of some phosphorylation sites of SrtA.** (**A**) The LC-MS/MS spectrum of phosphorylation on His-120 of SrtA. The phosphor-peptide GVSFAEENESLDDQNISIAG_p_HTFIDRPNYQFTNLK was observed at m/z 1013.71906. (**B**) The LC-MS/MS spectrum of phosphorylation on Asp-82 of SrtA. The phosphor-peptide VAGYIEIPDApDIKEPVYPGPATPEQLNR was observed at m/z 1045.50826. (**C**) The LC-MS/MS spectrum of phosphorylation on Thr-164 of SrtA. The phosphor-peptide DVKPpTDVGVLDEQK was observed at m/z 811.8862. (**D**) The LC-MS/MS spectrum of phosphorylation on Asp-160 of SrtA. The phosphor-peptide pDVKPTDVGVLDEQK was observed at m/z 811.3836. (**E**) The LC-MS/MS spectrum of phosphorylation on Lys-162 of SrtA. The phosphor-peptide DVpKPTDVGVLDEQK was observed at m/z 811.8862. Individual fragments are labeled based on the b- or y-ion nomenclature.

**References**

1. Bae T & Schneewind O (2006) Allelic replacement in Staphylococcus aureus with inducible counter-selection. *Plasmid* 55(1):58-63.

2. Ji Y, Marra A, Rosenberg M, & Woodnutt G (1999) Regulated antisense RNA eliminates alpha-toxin virulence in Staphylococcus aureus infection. *Journal of bacteriology* 181(21):6585-6590.

3. Sun F*, et al.* (2012) Protein cysteine phosphorylation of SarA/MgrA family transcriptional regulators mediates bacterial virulence and antibiotic resistance. *Proceedings of the National Academy of Sciences of the United States of America* 109(38):15461-15466.

4. Zhang J*, et al.* (2014) Antiinfective therapy with a small molecule inhibitor of Staphylococcus aureus sortase. *Proc Natl Acad Sci U S A* 111(37):13517-13522.

5. Duthie ES & Lorenz LL (1952) Staphylococcal coagulase; mode of action and antigenicity. *Journal of general microbiology* 6(1-2):95-107.

6. Kreiswirth BN*, et al.* (1983) The toxic shock syndrome exotoxin structural gene is not detectably transmitted by a prophage. *Nature* 305(5936):709-712.

7. Bae T*, et al.* (2004) Staphylococcus aureus virulence genes identified by bursa aurealis mutagenesis and nematode killing. *Proceedings of the National Academy of Sciences of the United States of America* 101(33):12312-12317.
